# Exploring Vietnamese co-authorship patterns in social sciences with basic network measures of 2008-2017 Scopus data

**DOI:** 10.12688/f1000research.12404.1

**Published:** 2017-08-24

**Authors:** Tung Manh Ho, Ha Viet Nguyen, Thu-Trang Vuong, Quang-Minh Dam, Hiep-Hung Pham, Quan-Hoang Vuong

**Affiliations:** 1 Institute of Philosophy , Vietnam Academy of Social Sciences, Hanoi , 100000, Vietnam; 2 Centre for Interdisciplinary Social Research, Western University Hanoi (ĐH Thành Tây), Hanoi, 100000, Vietnam; 3 Campus Européen de Dijon, Sciences Po Paris, Dijon, 21000, France

**Keywords:** Social network analysis, science collaboration, network characteristics, network visualization, research output.

## Abstract

**Background:** Collaboration is a common occurrence among Vietnamese scientists; however, insights into Vietnamese scientific collaborations have been scarce. On the other hand, the application of social network analysis in studying science collaboration has gained much attention all over the world. The technique could be employed to explore Vietnam’s scientific community.

**Methods:** This paper employs network theory to explore characteristics of a network of 412 Vietnamese social scientists whose papers can be found indexed in the Scopus database. Two basic network measures, density and clustering coefficient, were taken, and the entire network was studied in comparison with two of its largest components.

**Results:** The networks connections are very sparse, with a density of only 0.47%, while the clustering coefficient is very high (58.64%). This suggests an inefficient dissemination of information, knowledge, and expertise in the network. Secondly, the disparity in levels of connection among individuals indicates that the network would easily fall apart if a few highly-connected nodes are removed. Finally, the two largest components of the network were found to differ from the entire networks in terms of measures and were both led by the most productive and well-connected researchers.

**Conclusions:** High clustering and low density seems to be tied to inefficient dissemination of expertise among Vietnamese social scientists, and consequently low scientific output. Also low in robustness, the network shows the potential of an intellectual elite composed of well-connected, productive, and socially significant individuals.

## 1. Introduction

In early 2017, the Vietnamese public was once again disappointed to find out there was no Vietnamese universities in the Times Higher Education’s ranking of the top 300 universities in Asia. There was no shortage of experts’ attempts to explain this disappointing situation; many pointed to the fact that Vietnamese universities have not put enough focus on research. Being aware of the demand for improving research capacity, the Ministry of Education and Training has recently issued a number of policies and proposals addressing the issue head-on. Figuring among the many efforts is the issuance of circular No. 08/2017/TT-BGDĐT (issued on 14 ^th^ April, 2017) mandating doctoral students must have papers published in Scopus and Web of Science-indexed journals, the doctoral dissertation instructors must also have international publications. There has also been a proposal to mandate that candidates for the titles of Professor and Associate Professor must have international publications. Although these changes and proposals were met with both excitement and dread by the public, it is noteworthy that those who criticize the new regulations do not argue against the changes. Rather, their main concern is “when” or the timeline to adopt these policies: whether these changes are too abrupt.

In other words, people on both sides of the arguments express their desire to improve research capacity in Vietnam. The question remains is “how”: How to increase the quantity and quality of scientific publications in Vietnamese social sciences? The answer seems to be related to the spread of information and expertise in the scientific community, which may call for quantitative methods. However, the field of quantitative research on scientific activities and research policy in Vietnam is still nascent. Even though there have been several studies on the status of scientific publications in Vietnam, none has been carried out with a sole focus on social sciences – a field often criticized for having low productivity ^1, 2^. In addition, the technique of social network analysis is yet to be applied in the case of Vietnam, despite its potentials in explaining and predicting scientific performance. A study on the nature of scientific co-authorship among Vietnamese social scientists using network statistical analysis would yield valuable insights for policy-makers and educators in Vietnam.

### 1.1 Literature review

Over the years, the application of network statistical analysis on science collaboration has become pervasive; it has gleaned many insights into the dynamics of scientific activities as well as the properties of scholars’ networks. By exploring a number of databases from different fields such as biomedical research, physics and computer science, Newman showed that scientific collaboration networks seem to form “small worlds”, in which any randomly chosen pair of scientists would be separated only through a few intermediate collaborators. Another interesting aspect is that there are different degrees of clustering of scientists in different fields, suggesting the differences in social organizations ^3^. In a 2004 study of sociology collaboration networks by exploring of 30 years’ worth of data in the field, from 1963 to 1999, Moody discovered that participation in the network depends on the research major, and scholars who are more inclined to quantitative work are more likely to collaborate than those in non-quantitative work ^4^. In 2008, on the relationship between structural and socio-academic communities of co-authorship networks, Rodriguez and Pepe applied different community detection algorithms into the network of scholars in the field of wireless communication and sensors networks. They found out that even in interdisciplinary fields and multi-institutional research groups, co-authorship is heavily influenced by departments and institutional affiliations. In 2010, a study of network analysis on co-authorship and citation networks using topic-modelling path-finding algorithms showed that productive authors tend to cite and directly collaborate with colleagues sharing the same research interests ^5^.

Not only the application of network statistics is useful in characterizing the nature of scientist networks, it also provides a powerful tool to study and predict scientific performance such as productivity or research impact. A study on the effects of co-authorship on the performance of scholars using regression model and social network analysis showed that researchers who have a strong connection to only one co-author among a group of connected co-authors perform better than those who have many connections to the same group. The study also suggests it is possible to use professional social network of researchers to predict future performance ^6^. In 2013, a group of Taiwanese researchers examined co-authorship networks and research impact through social capital perspective. There are six indicators of social capital in the study: degree centrality, closeness centrality, betweenness centrality, prolific co-author count, team exploration, and publishing tenure. The team found that betweenness centrality is the most influential factor affecting citations of publications ^7^. Using data from library and information science in China, a Chinese research team constructed a network of co-authors, then compared an author’s centrality values with his/her citations. They found a high correlation between these two elements ^8^.

Meanwhile, in Vietnam, network statistics analysis has never been employed to study scientific activities. However, there have been a few attempts to study quantifiable aspects of scientific activities among Vietnamese scholars. Previous studies showed that Vietnam has a low scientific production rate in South East Asia, only equivalent to 13.33% of Singapore and 29% of Thailand in the period of 1991–2010 ^7, 9^. The total scientific output in Vietnam increased about 16 papers per year during the 1996–2001 period and increased by 20% from 2002 to 2010. It is worth noticing that the share of international collaboration was about 77% of the total publications, of which Japan was the largest collaborating country, followed by the United States, France, South Korea, and United Kingdom ^10, 11^. Furthermore, most of the key authors of these international projects did not come from Vietnam but from other countries (Manh 2015) ^10^. Mathematics was the only field where domestic output proportion was larger than the international. The largest segment was of biology and agriculture, in which 80–90% of published works involved inter-country collaborations. As for social sciences in Vietnam, a study on a sample of 412 Vietnamese scholars who have international publications in Scopus during the period of 2008–2017 has revealed that more than 90% of social scientists have published at least one co-written article (indexed in Scopus), and they worked in collaborations 13 times on average ^12^.

In short, faced with the current public desire to improve scientific output in social sciences in Vietnam, there is a shortage of in-depth quantitative analysis on the situation of information diffusion and of scientific output in the network of Vietnam scientists. Given the high frequency of co-authoring among social scientists in Vietnam, a network statistical analysis on collaboration among Vietnamese social scientists as the vector of connection would prove to be valuable. It would be interesting to see how network analysis – a technique first developed for studying networks in the natural world – yield valuable insights into the dissemination of knowledge and information of scientific nature among scholars in Vietnam.

### 1.2. Objectives of the study

This study aims to describe the basic properties of a co-authorship network in a sample of 412 Vietnamese social scientists who have published in Scopus-indexed journals and have online profiles, in the period of 2008–2017.

First, through analyzing the vertex degree distribution in the network, the study will discuss the concept of robustness of the network, which means how well-connected the network could remain if certain nodes and edges are removed. Then through the number of cliques and components, the study will describe the basic structure of the network. Furthermore, using metrics such as density and clustering coefficient, the status of the communication and exchange of scientific knowledge and expertise in the network will be analyzed.

Second, the study does not only provide numerical understanding of the network but also shows various ways in which it can be graphically represented. In doing so, the study will discuss the usefulness of several techniques of network graphical representation that can be applied to facilitate one’s understanding of the network.

Finally, the study will extract two of the largest components - one of the largest groups of connected scientists, then explore its characteristics. By comparing this component with the network of 412 Vietnamese social scientists, the study will provide deeper analysis on the concepts visited above.

## 2. Results

### 2.1. Characterizing the network of Vietnamese social scientists

**Figure 1.**
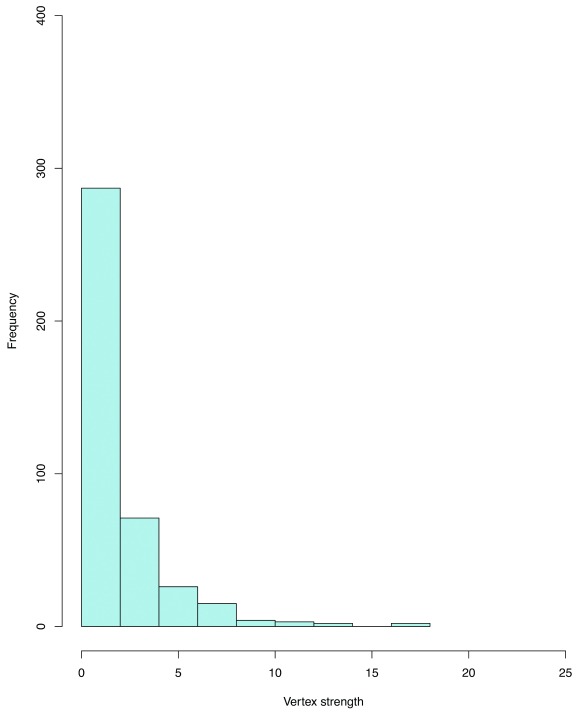
A histogram of vertex degree distribution. This is a histogram of vertex degree distribution of the full 412-node network. The degree of each node is measured as the number of co-authored papers, or connections, of each individual in the dataset.

Using R, the dataset employed in this paper counts 412 vertices in the Nodes list and 401 edges in the Edges list. Each vertex or node can be different in terms of degree. The average vertex degree is 1.95 with standard deviation 2.26. This means on average, one Vietnamese social scientist co-authors with about two other Vietnamese authors. Figure 1 visualizes the distribution of vertex degrees and shows the disparity between the least and most well-connected authors. ( Figure 1 can be plotted using the command in Supplementary File 1 “Rcommands_fig1.doc”.)

An overwhelming majority of researchers - about 280 out of 412 - possesses degree from 0 to 2; only about 50 researchers have a vertex degree of 3-4, and the number of authors with higher degree decreases dramatically from degree 4 upwards. In other words, most researchers in Vietnam have less than two connections – less than two co-authored papers – and only very few has more than four. Clearly, rather than being composed of mostly people with the same level of connections, the network consists of a few very well-connected people, while the rest does not have many connections at all. It can be inferred that it would be possible to break the network into multiple components if we just removed those few well-connected nodes (people of degree higher than 5) or their links. In network analysis literature, how well-connected a network remains when some vertices and edges are taken out is referred to as robustness ^13^. Thus, in this study, the degree distribution reveals that the network of Vietnamese social scientists is not robust. This effect can be seen more visibly when we explore the characteristics of one of the biggest components of this network.

To explore the structure and cohesiveness of the network, it is useful to look at censuses of cliques, components, graph density, and transitivity (Commands for calculation of the network metrics can be found in Supplementary File 2 “Rcommands_metrics.doc”).

By generating a census of cliques of all sizes, we can get a general sense of the structure of the network:

As shown in Table 1, in this network, there are 412 nodes (clique of size 1), 401 edges (clique of size 2), 281 triangles (cliques of size 3), 201 cliques of size 4, and so on. The largest clique is size 9, of which there is only one.

**Table 1.** A census of cliques of all sizes for the network of Vietnamese social scientists. A clique is a subset of vertices that are fully cohesive, meaning that all vertices are connected by one link. This table lists all clique sizes that exist within the dataset and the number of cliques in each size category.

**Clique size**	1	2	3	4	5	6	7	8	9
**Numbers**	412	401	281	201	144	86	36	9	1

A graph is considered to be connected if every node could be reached by any other node (i.e. if for any two nodes, there is a walk between the two). Looking at Table 2, we can see that the network of Vietnamese social scientists is not connected; there are 125 components of size 1. About 30% of the scientists in this study are isolated nodes in the network, possibly because they either work alone or work exclusively with foreigners. Alternatively, the five biggest components (size 11, 15, 16, 27 and 43) together takes up another 30%, while the rest consists of all middle-sized components (size 2–9).

**Table 2.** A census of components of all size for the network of Vietnamese social scientists. A component is a subgraph in which every vertex can be reached from every other, no matter how many links constitute the path. This table lists all component sizes that exist within the dataset and the number of components in each size category

**Component size**	1	2	3	4	5	6	7	9	10	11	15	16	27	43
**Numbers**	125	24	9	3	4	4	2	1	1	2	1	1	1	1

By calculating the density and transitivity of the graph, it can be seen that the network is very sparse. The density of the graph is 0.0047, indicating only about 0.47% of potential edges are realized in this network. On the other hand, when three vertices are connected at all, there is a better than a 50/50 chance they will form a triangle (clique of size 3): The global clustering coefficient of the collaboration graph is 0.5862, indicating that nearly 59% of connected triples have formed triangles. Given that there is a clear relationship between the speed of the spread of information and clustering coefficient; the higher the clustering coefficient, the slower the information spread ^14^, it is reasonable to assume when two scientists co-author in a scientific paper, there is a great deal of knowledge and expertise to be communicated and exchanged. Hence, the low density and high clustering coefficient of the network suggests that the dissemination of knowledge and expertise among 412 Vietnamese social scientists in this study is not happening as smoothly as possible.

### 2.2. Network visualization

Visual representations of the network is done through figure plotting in R. Commands for data set-up required for figure plotting can be found in Supplementary file 3 “Rcommands_graph.doc”.

There are several ways to visually represent the network. Here, the study aims to strike a balance between creating a graph both visually attractive and useful in facilitating the statistical understanding of the previous histogram and analysis.

**Figure 2.**
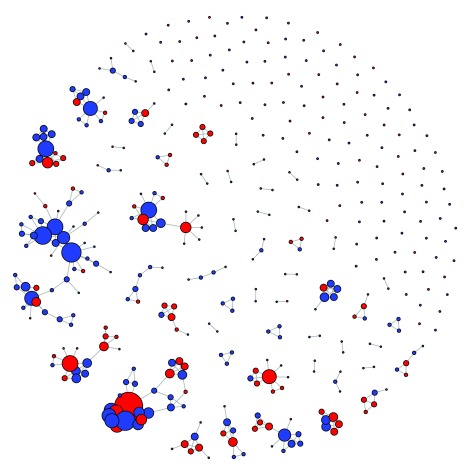
A visual representation of the network of 412 Vietnamese social scientist. This figure is a visualization of the full 412-node network in Fruchterman-Reingold layout. Nodes are color-coded based on author gender (blue for male, red for female). Node sizes are based on node degrees. Edges are represented by a line connecting concerned nodes.

Figure 2 was conceived as a primary representation of the network, highlighting vertex degree, density, transitivity, and robustness using various visual cues. Among many attributes of the nodes that have been collected (region, age, title, etc.), biological gender has been chosen as the basis because of its relatively simple binary nature. In this study, blue color represents male and red represents female. Such simplicity is hoped to make the graph more aesthetically appealing. Meanwhile, the size of each vertex is determined by the number of edges incident on each node – in other words, by the vertex degree. Hence, the higher the number of edges incident upon a vertex, the bigger the vertex is. This is to make visible the gap between the well-connected scientists and the more isolated ones, one of the most striking features of the network as shown in section 2.1. For layouts, among all those available in R(v3.1.1)’s igraph packages, layout Fruchterman-Reingold is chosen because it makes the structure of the network nicely perceptible: 30% of nodes fall into five largest components, 40% are middle-size components, and the 125 left are isolated nodes (recall the statistics on components in section 2.1). Commands for plotting this figure can be found in the Supplementary File 4 (“Rcommands_fig2.doc”).

**Figure 3.**
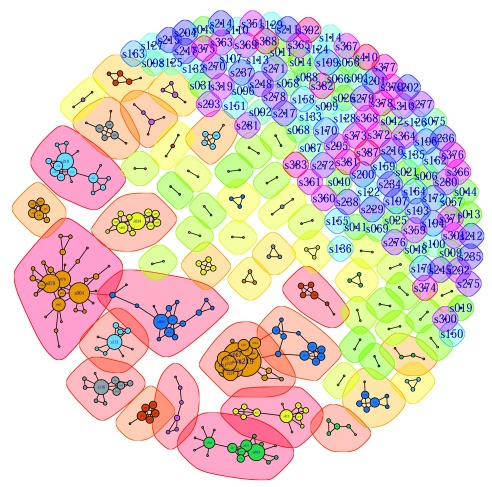
Network visualization with community detection algorithm. This figure is a visualization of the full 412-node network in Fruchterman-Reingold layout with community detection. Potential communities are partitioned using colored regions with boundaries. Colors are mostly to facilitate visual perception and irrelevant to the understanding of the data.

Seeking more insights on the network, a community detection algorithm was run on the data, which resulted in Figure 3, a second visualization that complemented Figure 2. (This can be performed using the commands provided in Supplementary File 5 “Rcommands_fig3.doc”.) Looking at the biggest components in Figure 3, one can see a new pattern emerges: though the big components are fully connected, they do not seem to be one big close group; rather, they seem to consist of a few smaller communities of very closely connected scientists, and these communities are linked together by one or two vertices acting as weak links. The algorithm does indeed break the two big groups into smaller communities with one or two vertices that connect these communities.

In the next section, the two largest components, component size 43 and component size 27, will be studied more in-depth.

### 2.3. Exploring the characteristics of the two largest components

Recall that component is a technical term in network theory that refers to a maximally connected subgraph, in which any two vertices can be reached from another via a path consisting of any number of edges and nodes. Thus, any graph can be constituted by many different components. In this study, the network of 412 Vietnamese social scientists is the sum total of 179 components of various size, ranging from 1 to 43; the two largest components have 43 and 27 nodes each. One can treat such components as independent networks in and of themselves. In this section, the characteristics of these two largest components will be explored and compared with the whole network. From this point on, the components will be called Comp43 and Comp27, and the original network will be dubbed Net412. As one might expect, as we zoom in, there will be differences in the properties of the components in question and that of the network as a whole. Table 3 summarizes and compares the basic metrics of Comp43, Comp27 and Net412.

**Table 3.** Comparison of basic network metrics of Net412, Comp43 and Comp27. Vertex degree is the number of edges incident upon a vertex. Density is the frequency of realized edges (connections) relative to potential edges (connections). Transitivity (or clustering coefficient) is the relative frequency with which connected triples of vertices form triangles. Net412 is the full 412-node network consisting of the entire dataset. Comp43 and Comp27 are the 43-node and 27-node components, respectively, which are subsets in which every vertex can be reached by every other.

Metrics	Net412	Comp43	Comp27
**Graph density**	0.47%	7.20%	22.51%
**Mean degree**	1.95	3.02	5.58
**Transitivity**	58.62%	32.43%	70.43%
**Mean total publications**	3.56	5.53	2.00

In all network metrics, Comp27 scores the highest. Specifically, in terms of density of connections, Net412 is the sparsest, 0.47%. The density of Comp43 (7.20%) is 14-fold that of Net 412, and the same characteristic in Comp27 (22.51%) is 44-fold compared to that of the whole network. Regarding average vertex degree, Comp27 is the highest followed by Comp43 then Net412. Concerning global clustering coefficient (or transitivity), Comp27 towers over Net412 by 11 percentage points (70% versus 59%), while the latter is in turn over 2 times higher than Comp43 (70% versus 32%).

High clustering and low density suggest a certain level of inefficiency in the spread of knowledge and expertise (as explained in section 1.1 on the characteristics of the network of 412 Vietnamese social scientists); either could be the cause of the other. Thus, from the network metrics, one would expect Comp27’s dissemination of scientific knowledge and expertise to be less efficient than Comp43. In fact, even though the density of connection in Comp27 is about 3 times that of Comp43, its effects would be limited because of the higher clustering. One can then ask how to verify that high clustering cancels the good effects of even high density. Supposing that better dissemination of scientific knowledge and expertise can be observed in a better scientific output, we could look at the mean value of total publications of scientists in each network for insights on the aforementioned question. Indeed, as Table 3 shows, Comp43 performs better than Comp27 in terms of scientific output – almost 3 times higher, 5.53 versus 2.00.

**Figure 4.**
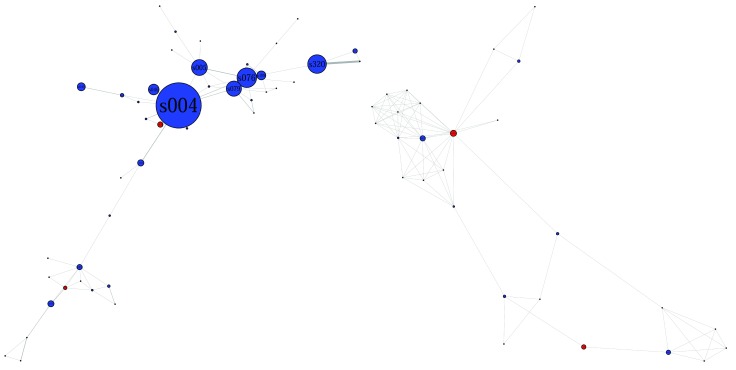
Visualization of Comp43 (left) and Comp27 (right) with node size equals scientific output. This figure is a visualization of the full 43-node and 27-node components in Fruchterman-Reingold layout. Nodes are color-coded based on author gender (blue for male, red for female). Node sizes are based on node degrees. Edges are represented by a line connecting concerned nodes.

The difference in scientific output between Comp43 and Comp27 can be viewed in Figure 4 below. Commands for plotting Figure 4 (left and right) can be found in Supplementary File 6 and Supplementary File 7 (“Rcommands_fig4left.doc” and “Rcommands_fig4right.doc” respectively.)

**Figure 5.**
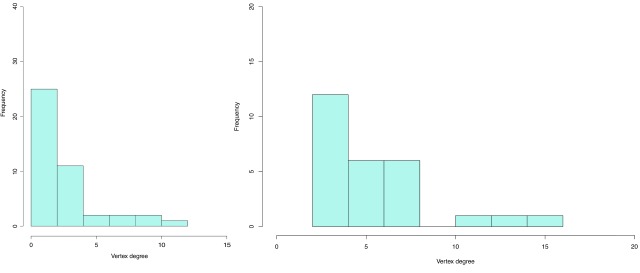
Histogram of degree distribution of Comp43 (left) and Comp27 (right). These are histograms of vertex degree distribution of the 43-node and 27-node components. The degree of each node is measured as the number of co-authored papers, or connections, of each individual in the dataset.

Besides revealing the differences in scientific output of the two networks, Figure 4 also reveals that nodes in both networks seems to revolve around one or two important nodes with higher level of scientific output. In Comp43, it is node s004 and in Comp27, it is node s067 and s219 (the visible blue and red dots on the left side of Figure 4). It is interesting that these three nodes have highest numbers of edges incident upon them in their respective networks; s004 has a degree of 11, highest in Comp43; s067 has a degree of 13 and s319 has a degree of 16, also highest in Comp27. If these important vertices are to be removed, the networks would break apart into several smaller components. This feature was referred to in section 5.1 through the concept of robustness, and it should be noted that Net412 is not robust. The situation is the same for Comp43 and Comp27. In Figure 5, the histogram distributing the degrees of nodes in these networks shows a clear disparity in vertex degree.

Commands for plotting Figure 5 (left and right) in R can be found in Supplementary File 8 and Supplementary File 9 (“Rcommands_fig5left.doc” and “Rcommands_fig5_right.doc” respectively).

## 3. Discussion

After performing social network analyses on a sample of 412 social scientists in Vietnam, whose information has been gathered primarily from their Scopus profiles.

First, the study has shown that the network has a low level of connection with only 0.47% of all potential edges realized, and high in clustering with 59% chance a connected triple would close into a triangle. These two characteristics together suggest a reality that the communication and exchange of knowledge and expertise among the Vietnamese social scientists are not very efficient. In addition, the degree distribution reveals that it would be difficult for the network to stay well-connected when a few highly-connected nodes and their edges are removed; or, in network theory’s terminology, the network is not very robust.

Second, in this study, network visualization is shown to be useful not only in facilitating quantitative understanding but also in discovering new insights into the structures of the network. By applying appropriate techniques of graph plotting, the disparity of the level of connections and the structure of the network can be easily visualized. Using the community detection algorithm, an interesting fact about these biggest groups is unraveled: they mostly comprised of smaller and tightly connected communities with one or two vertices connecting these altogether.

Third, close investigations show that the two largest components in the network have different characteristics from the 412-node-graph. Both smaller networks have more connections than the big one, but in terms of clustering, the 43-node-graph has a much higher level of clustering. Despite these differences, all the three networks resemble in low level of robustness and high disparity in terms of degree distribution, which means when the most connected people are removed from the networks, these latter would immediately be decomposed into several smaller groups. Most strikingly, the two smaller networks seem to be led by the most productive researchers in them, who also have the most connections.

Given the mostly high transitivity of all three networks, it could be remarked that the original 412-node network could be considered more or less a sum of smaller communities centered around well-connected nodes. On a more ego-centric and contextual note, there seems to be a relationship between the social status (their position in an institution, for example) of an individual in the network and his or her importance to the network (whether he/she has the most connections or being central to many connections in some ways) as well as his or her scientific output, as suggested by the examples of node s004, s067 and s219. These individuals are few and far between in a network of high disparity in vertex degree, and present a stark contrast with their peers in terms of both connections and productivity. They have the potentials to form a group of intellectual elites.

Finally, there is still much to be learned from both the dataset of 412 social scientists and the network that can be constructed from the raw data. For example, though the study has hinted at the difference in scientific output of two networks (comparison of Comp43 and Comp27 in section 2.3), it is worth considering a more systematic examination of the relationship between a network’s properties and the scientific output of the vertices it contains. Thus, finding out whether a correlation among these variables exists does merit further investigation. Another promising area of research is the exploration of diversity in scientific co-authorship. In this study, node color is coded by gender (section 2.2), but other attributes such as age, region, work, titles, etc. can also be added to the analysis as well.

This paper cannot claim to have exhausted the toolkits that social network analysis could provide. There are still many other aspects of the network worthy of further investigation. How would the network turn out if other dimensions such as weights or durability of the relational data are added to the analysis? How useful are certain aspects of the network in predicting scientific performance? How would this network evolve over time? Not only intellectually stimulating, these important questions are of tremendous practical value for policy-makers and educators, particularly when their decision-making concerns education policies and research organizations. Further investigation in this area of research and on this topic is thus necessary.

## 4. Materials and methods

### 4.1. Materials: Original data and the network data set

The data for this study was derived from a dataset on the productivity of Vietnamese scientists in the field of social sciences collected by Vuong & Associates. The investigation, which took place within two months from March to April 2017, was conducted under the license V&A/03/2017, issued on 15 March, 2017.

First, we constructed a file that contains data on all the attributes of each author, called a “Nodes list” ( Dataset 1: "20170725_net412_ NODES.csv"). The data collection process was monitored regularly to ensure its reliability, including the following steps: first, the research team used sources such as personal and institutional websites of authors, websites of journals where their works were published, Google Scholar, and Scopus database to collect data. Then, to check the accuracy of the information, we compare various online sources where each author’s information can be found; for example, Google scholar versus Scopus, personal websites versus institutional websites. After this process, the research team obtained a complete dataset of 412 scholars’ information, consisting of: (i) age, sex, region; (ii) affiliations; (iii) fields of study; (iv) the number of publications in Scopus, (v) the number of research years since the Master graduation; (vi) the number of researchers they collaborated with; (vii) whether or not they have the title of “Professor/Assoc. Professor”. All of this essentially constitutes the node.

**Figure 6.**
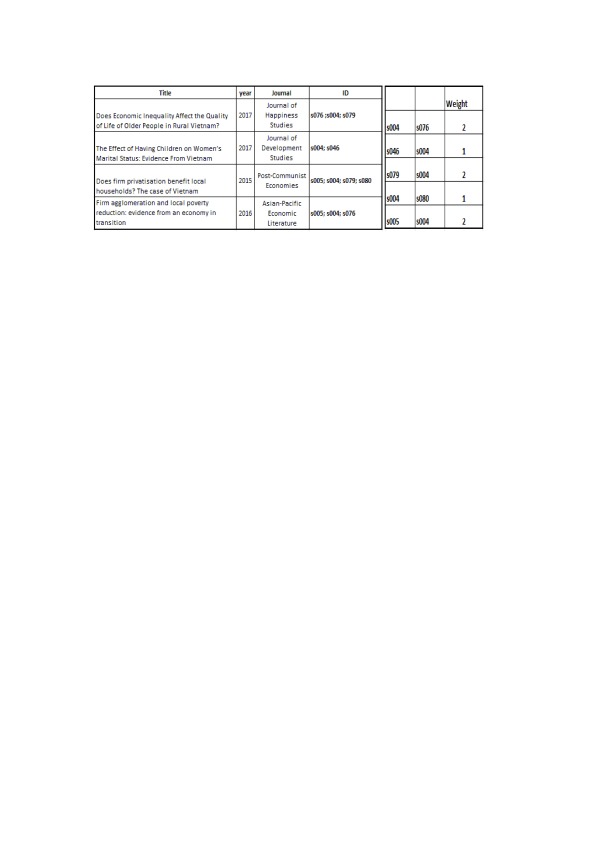
An example of the process of handling relational data. In these figures, a fraction of the construction of Edges lists is shown. The table on the right shows how we record 4 published articles in which 5 Vietnamese scientists coded as s004, s005, s076, s079, s080 take part as co-authors. The table on the right shows every pair that have collaborated at least once among these 5 scientists, as well as the number of collaborations of each pair, which are considered the “weight” of the relation.

Based on this information, we then construct our relational data, which is called an “Edges list” ( Dataset 2: "20170729_net412_LINKS.csv"). We consider two authors as exhibiting a co-authorship tie when they appear together in a scientific publication. Each time the same two authors appear together in a paper, it is counted toward the “weight” of the tie. The example of an edges list can be seen in the following figure. The data was then processed and analyzed using statistical software R (v3.3.1). Figure 6 shows an example of how relational data is handled in the study. To illustrate, in the first row of the table on the left side, a published paper being co-authored by scientists ID s004, s076 and s079 is recorded into the database first. Then on the right side, co-authorship relations among these three scholars are recorded; and the weight is the count of how many times each pair co-authors.

The data for Comp43 and Comp27 were manually extracted from the full dataset. Nodes lists ( Dataset 3 “20170719_comp43_NODES.csv” and Dataset 5 "20170726_comp27_NODES.csv") and Links lists ( Dataset 4 “20170719_comp43_LINKS.csv” and Dataset 6 "20170729_comp27_LINKS.csv") for Comp43 and Comp27 respectively were constructed by picking relevant edges and nodes from the original lists.

Dataset 1. 20170725_net412_ NODES.csv This dataset contains all 412 individuals in the study and their attributes. Each individual is considered a node (vertex) in the network. Click here for additional data file. Copyright: © 2017 Ho TM et al. 2017 Data associated with the article are available under the terms of the Creative Commons Zero "No rights reserved" data waiver (CC0 1.0 Public domain dedication).

Dataset 2. 20170729_net412_LINKS.csv This dataset lists the number of co-written articles between all 412 authors of the network, where relevant. Each collaboration is counted as a link (edge) in the network. Click here for additional data file. Copyright: © 2017 Ho TM et al. 2017 Data associated with the article are available under the terms of the Creative Commons Zero "No rights reserved" data waiver (CC0 1.0 Public domain dedication).

Dataset 3. 20170719_comp43_NODES.csv This dataset contains 43 individuals in the 43-node component and their attributes. Each individual is considered a node (vertex) in the component Click here for additional data file. Copyright: © 2017 Ho TM et al. 2017 Data associated with the article are available under the terms of the Creative Commons Zero "No rights reserved" data waiver (CC0 1.0 Public domain dedication).

Dataset 4. 20170719_comp43_LINKS.csv This dataset lists the number of co-written articles between the 43 authors of the 43-node component, where relevant. Each collaboration is counted as a link (edge) in the component. Click here for additional data file. Copyright: © 2017 Ho TM et al. 2017 Data associated with the article are available under the terms of the Creative Commons Zero "No rights reserved" data waiver (CC0 1.0 Public domain dedication).

Dataset 5. 20170726_comp27_NODES.csv This dataset contains 27 individuals in the 27-node component and their attributes. Each individual is considered a node (vertex) in the component. Click here for additional data file. Copyright: © 2017 Ho TM et al. 2017 Data associated with the article are available under the terms of the Creative Commons Zero "No rights reserved" data waiver (CC0 1.0 Public domain dedication).

Dataset 6. 20170729_comp27_LINKS.csv This dataset lists the number of co-written articles between the 27 authors of the 27-node component, where relevant. Each collaboration is counted as a link (edge) in the component. Click here for additional data file. Copyright: © 2017 Ho TM et al. 2017 Data associated with the article are available under the terms of the Creative Commons Zero "No rights reserved" data waiver (CC0 1.0 Public domain dedication).

### 4.2. Methods of Analysis

The method employed in this study was statistical analysis of network data. There were several reasons why we choose this method. First, the prevalence of co-authorship in research efforts among Vietnamese scientists as shown in the literature review naturally prompts us to ponder on how the co-authors cooperate and the kinds of interactions that exist among them. Second, as we find out that social network analysis has been applied widely all over the world in the study of scientific collaborations, we expect a match between our interest in characterizing collaboration among Vietnamese social scientists and the technical tools this approach provide. Finally, the help of statistical software allows us to create graphic representation of the network, which supplements all the rigorous numerical analysis with a more intuitive way of understanding interactions among actors in the network.

In this study, we will only focus on a descriptive analysis of our network data. The study is strictly limited to the interactions among Vietnamese scholars only. There are two caveats with regards to the method and the scope of the analysis. First, as the collaborations with foreign scholars are not accounted for in this study, certain interesting features of the networks can be lost. For example, a foreign scholar could cooperate with two Vietnamese scholars, but these Vietnamese scholars might not publish together. Thus, a link is missing. The cumulative effects of this kind of missing links can make the network appear much less connected than it actually is. Second, network analysis is first developed to solve problems in areas such as mathematics, chemistry, electrical circuits, operational research, and computer science before being applied by sociologists in mid-20th Century to study social network, hence, we can expect there are inherent limits to the explanatory power of the technique.

### 4.3. Network characterizations

In order to understand the visualization of a network, it is important to familiarize oneself with the terminologies of statistical network analysis. Here, we provide an explanation of terms that are relevant for the scope and purpose of this paper. More technical explanations of the terms in this paper can be found in *Statistical analysis of network data with R* ^15^, and *Social Network Analysis: A Handbook, Second edition* ^16^.

A graph *G*= ( *V*, *E*) is a mathematical structure consisting of a set *V* of vertices (or nodes) and a set *E* of edges (or links); elements of *E* are links between a pair of distinct vertices belongs to set *V*. When two nodes are connected to each other by an edge, they are said to be adjacent. In this study, a vertex represents a Vietnamese social scientist, which means the total number of vertices is 412. An edge represents a relationship between two distinct Vietnamese social scientists. A concept that connects edge and vertex is degree; a degree of a vertex is the counts of the number of edges incident upon that vertex. For instance, if there are three edges incident upon a vertex, the degree of that vertex is three.

Notice that depending on the attribute of the relationships between two vertices, an edge might or might not have a direction, thus there might be a need to specify the ordering of the pair of vertices in each edge in set *E*. A directed graph is a graph where each edge in *E* has an ordering to its vertices; an undirected graph is a graph where an edge needs not to be defined by the ordering in the vertices. In this study, since the relationship among co-authors is considered to be neutral, the graph that shows their relational ties will be undirected.

To understand the structure of a network, two fundamental concepts are clique and component. A clique is a subset of vertices that are fully cohesive, in that, all vertices within this subset are connected by edges. For example, a node is a clique of size one, an edge is a clique of size two, a triangle is a clique of size three, and so on. A component is a subgraph, in which, every vertex can be reached from every other. It is easy to see the different between a clique and a component. In a clique, every two nodes must be connected by an edge or in other words, they must be adjacent; while in a component, every two nodes might or might not be connected by an edge, but they must be somehow connected through a path consisting of a number of other edges and nodes.

Regarding the structure of a network, it is natural to wonder about the level of cohesion of the network: How frequent do the edges appear? How likely do three connected nodes close into a clique size 3? These questions can be answered using the concept of density and global clustering coefficient, also known as transitivity. The density of a graph is the frequency of realized edges relative to potential edges. It can be calculated using the following formula:

                                                                                                              density = 2 *l*/[ *n*( *n*-1)]                    

in which *l* is the numbers of links (or edges), and n is the number of nodes (or vertices). The clustering coefficient (or transitivity) measures the relative frequency with which connected triples of vertices form triangles:

*cl*_T_(G) = 3 *τ*Δ(G)/ *τ*_3_(G)                   

in which *τ*Δ(G) is the number of triangles in the graph G; and *τ*_3_(G) the number of subgraphs consist of three vertices connected by two edges, i.e. connected triples.

Armed with understanding of relevant technical concepts, we are able to explore the characteristics of the network of 412 Vietnamese social scientists.

## 5. Conclusions

With the purpose of understanding the structure and characteristics of the network of 412 Vietnamese social scientists, the study has applied the technique of social network analysis to give a sense of the structure of the network, the level of connection as well as the level of clustering in the network. In the last parts of this paper, we zoomed into the two largest components of the network and compare their relevant characteristics together with the network of the entire sample (in line with the spirit of 17).

Remarks corresponding to each characteristic along with insights into the robustness of the network and the spread of scientific knowledge and expertise in the network have been extracted and discussed. The high clustering of the entire network of 412 Vietnamese social scientists and low density shared by both the original network and its two component networks, seem to be closely related to inefficient dissemination of academic expertise. Both of these in turn lead to modest scientific output, which is at the heart of the perpetual discussions on research capacity in Vietnam. Furthermore, the network, low in robustness, is only held together by a few well-connected scholars, who seem to also hold significant social positions. This suggests the existence of certain intellectual elites who could perhaps propel Vietnamese scientific output.

## Data Availability

The data referenced by this article are under copyright with the following copyright statement: Copyright: © 2017 Ho TM et al.

Data associated with the article are available under the terms of the Creative Commons Zero "No rights reserved" data waiver (CC0 1.0 Public domain dedication). 

Dataset 1: "20170725_net412_NODES.csv" This dataset contains all 412 individuals in the study and their attributes. Each individual is considered a node (vertex) in the network. 10.5256/f1000research.12404.d174929 ^18^

Dataset 2: "20170729_net412_LINKS.csv" This dataset lists the number of co-written articles between all 412 authors of the network, where relevant. Each collaboration is counted as a link (edge) in the network. 10.5256/f1000research.12404.d174930 ^19^

Dataset 3: “20170719_comp43_NODES.csv” This dataset contains 43 individuals in the 43-node component and their attributes. Each individual is considered a node (vertex) in the component. 10.5256/f1000research.12404.d174931 ^20^

Dataset 4: “20170719_comp43_LINKS.csv” This dataset lists the number of co-written articles between the 43 authors of the 43-node component, where relevant. Each collaboration is counted as a link (edge) in the component. 10.5256/f1000research.12404.d174932 ^21^

Dataset 5: "20170726_comp27_NODES.csv" This dataset contains 27 individuals in the 27-node component and their attributes. Each individual is considered a node (vertex) in the component. 10.5256/f1000research.12404.d174933 ^22^

Dataset 6: "20170729_comp27_LINKS.csv" This dataset lists the number of co-written articles between the 27 authors of the 27-node component, where relevant. Each collaboration is counted as a link (edge) in the component. 10.5256/f1000research.12404.d174934 ^23^

## Supplementary Materials

Supplementary File 1: Commands for plotting Figure 1: “Rcommands_fig1.doc” This file contains the R command used to plot the histogram of vertex degree distribution of the full 412-node network shown in Figure 1.

Supplementary File 2: Commands for computing network metrics: ”Rcommands_metrics.doc” This file contains the commands that compute network metrics.

Supplementary File 3: Commands for graph plotting: “Rcommands_graph.doc” This file contains the commands that set up the data for graph plotting in R.

Supplementary File 4: Commands for plotting Figure 2: “Rcommands_fig2.doc” This file contains the R commands used to plot the visualization of the full 412-node network in Fruchterman-Reingold layout shown in Figure 2.

Supplementary File 5: Commands for plotting Figure 3: “Rcommands_fig3.doc” This file contains the R commands used to run a community detection algorithm on the full 412-network and visually present in on the base of Figure 3.

Supplementary File 6: Commands for plotting Figure 4 – left: “Rcommands_fig4left.doc” This file contains the R commands used to plot the visualization of the 43-node component shown in Figure 4 (left).

Supplementary File 7: Commands for plotting Figure 4 – right: “Rcommands_fig4right.doc” This file contains the R commands used to plot the visualization of the 27-node component shown in Figure 4 (right).

Supplementary File 8: Commands for plotting Figure 5 – left: “Rcommands_fig5left.doc” This file contains the R commands used to plot the histogram of vertex degree distribution of the 43-node component shown in Figure 5 (left). These commands should be used in continuation with the data set-up from “Rcommands_fig4left.doc”.

Supplementary File 9: Commands for plotting Figure 5 – right: “Rcommands_fig5right.doc” This file contains the R commands used to plot the histogram of vertex degree distribution of the 27-node component shown in Figure 5 (right). These commands should be used in continuation with the data set-up from “Rcommands_fig4right.doc”.
