# Supplementary material for: Exploring Vietnamese co-authorship patterns in social sciences with basic network measures of 2008-2017 Scopus data
Source: F1000Res. 2017 Aug 24;6:1559. [Version 1] doi: 10.12688/f1000research.12404.1 (PMC5590089; doi:10.12688/f1000research.12404.1)
Supplement: Supplementary file 8 [file f1000research-6-13433-s0007.tgz › e0a17d61-2221-4639-be0f-913701ec4f47.doc]

Commands in R for computing network metrics

nodes <- read.csv("C:/.../20170725_net412_NODES.csv",header=T, as.is=T)

links <- read.csv("C:/.../20170729_net412_LINKS.csv", header=T, as.is=T)

library(igraph)

net <- graph_from_data_frame(d=links, vertices=nodes, directed=F)

net <- simplify(net, remove.multiple = T, remove.loops = T)

vcount(net)

ecount(net)

graph.density(net)

transitivity(net)

mean(degree(net))

sd(degree(net))

table(sapply(cliques(net),length))

comps<-decompose.graph(net)

table(sapply(comps,vcount))
