# Supplementary material for: Exploring Vietnamese co-authorship patterns in social sciences with basic network measures of 2008-2017 Scopus data
Source: F1000Res. 2017 Aug 24;6:1559. [Version 1] doi: 10.12688/f1000research.12404.1 (PMC5590089; doi:10.12688/f1000research.12404.1)
Supplement: Supplementary file 9 [file f1000research-6-13433-s0008.tgz › e5f2f3ea-08df-4edb-ae41-b16709774102.doc]

Commands in R for graph plotting

nodes <- read.csv("C:/.../20170725_net412_NODES.csv",header=T, as.is=T)

links <- read.csv("C:/.../20170729_net412_LINKS.csv", header=T, as.is=T)

head(links)

nrow(nodes); length(unique(nodes$id))

nrow(links); nrow(unique(links[,c("from","to")]))

links <- aggregate(links[,3], links[,-3], sum)

library(igraph)

net <- graph_from_data_frame(d=links, vertices=nodes, directed=F)

class(net)

E(net)

V(net)

net <- simplify(net, remove.multiple = T, remove.loops = T)

plot(net, layout=layout.kamada.kawai(net), edge.arrow.size=.1,vertex.size=3,vertex.label=NA)

a=read.csv("C:/.../20170725-Socsci-NODES.csv",header=T, as.is=T)

V(net)$Sex=as.character(a$sex[match(V(net)$name,a$id)])

V(net)$Sex

V(net)$color=V(net)$Sex

V(net)$color=gsub("M","blue",V(net)$color)

V(net)$color=gsub("F","red",V(net)$color)

nodesize=degree(net)

plot(net, layout=layout.kamada.kawai(net), edge.arrow.size=.105, vertex.size=nodesize, vertex.label=NA)

hist(degree(net),col="lightblue",xlim=c(0,25),ylim=c(0,400),xlab="Vertex degree", ylab="Frequency", main="")
