# Supplementary material for: Exploring Vietnamese co-authorship patterns in social sciences with basic network measures of 2008-2017 Scopus data
Source: F1000Res. 2017 Aug 24;6:1559. [Version 1] doi: 10.12688/f1000research.12404.1 (PMC5590089; doi:10.12688/f1000research.12404.1)
Supplement: Supplementary file 10 [file f1000research-6-13433-s0009.tgz › dac6dc77-e941-46ab-a81b-18e0d59291d2.doc]

Commands in R for plotting Figure 2

net <- graph_from_data_frame(d=links, vertices=nodes, directed=F)

net <- simplify(net, remove.multiple = T, remove.loops = T)

a=read.csv("C:/…/20170725_net412_NODES.csv",header=T, as.is=T)

V(net)$Sex=as.character(a$sex[match(V(net)$name,a$id)])

V(net)$color=V(net)$Sex

V(net)$color=gsub("M","blue",V(net)$color)

V(net)$color=gsub("F", "red", V(net)$color)

nodesize=degree(net)

plot(net, layout=layout_with_fr(net),vertex.size=nodesize*0.8,vertex.label=NA, edge.color="darkgray")
