# Supplementary material for: Exploring Vietnamese co-authorship patterns in social sciences with basic network measures of 2008-2017 Scopus data
Source: F1000Res. 2017 Aug 24;6:1559. [Version 1] doi: 10.12688/f1000research.12404.1 (PMC5590089; doi:10.12688/f1000research.12404.1)
Supplement: Supplementary file 11 [file f1000research-6-13433-s0010.tgz › a6c26119-97f5-468c-8086-878560d5d3d3.doc]

Commands in R for plotting Figure 3

netc<-fastgreedy.community(net)

plot(netc, net, edge.arrow.size=.105,vertex.size=nodesize*0.85,vertex.label.cex=nodesize*0.04)
