# Supplementary material for: Exploring Vietnamese co-authorship patterns in social sciences with basic network measures of 2008-2017 Scopus data
Source: F1000Res. 2017 Aug 24;6:1559. [Version 1] doi: 10.12688/f1000research.12404.1 (PMC5590089; doi:10.12688/f1000research.12404.1)
Supplement: Supplementary file 13 [file f1000research-6-13433-s0012.tgz › 7c2c42ce-1a9f-41ed-8dfd-26726a47e77c.doc]

Commands in R for plotting Figure 4 (right – Comp27)

nodes <- read.csv("C:/…/20170726_comp27_NODES.csv",header=T, as.is=T)

links <- read.csv("C:/…/20170729_comp27_LINKS.csv", header=T, as.is=T)

head(links)

nrow(nodes); length(unique(nodes$id))

nrow(links); nrow(unique(links[,c("from","to")]))

links <- aggregate(links[,3], links[,-3], sum)

library(igraph)

net <- graph_from_data_frame(d=links, vertices=nodes, directed=F)

net <- simplify(net, remove.multiple = T, remove.loops = T)

a=read.csv("C:/…/20170726_comp27_NODES.csv",header=T, as.is=T)

V(net)$Sex=as.character(a$sex[match(V(net)$name,a$id)])

V(net)$color=V(net)$Sex

V(net)$color=gsub("M","blue",V(net)$color)

V(net)$color=gsub("F", "red", V(net)$color)

nodesize=nodes$ttlitems

plot(net, layout=layout_with_fr(net), vertex.size=nodesize*0.5, vertex.label.cex=nodesize*0.04,	vertex.label.color ="black", edge.width=links$x*0.3, edge.color="darkgray")
