# Supplementary material for: Exploring Vietnamese co-authorship patterns in social sciences with basic network measures of 2008-2017 Scopus data
Source: F1000Res. 2017 Aug 24;6:1559. [Version 1] doi: 10.12688/f1000research.12404.1 (PMC5590089; doi:10.12688/f1000research.12404.1)
Supplement: Supplementary file 15 [file f1000research-6-13433-s0014.tgz › 3c29634e-31c6-4998-82b5-625611185746.doc]

Commands in R for plotting Figure 5 (right – Comp27)

hist(degree(net),col="lightblue", xlim=c(0,20),ylim=c(0,20), xlab="Vertex degree", ylab="Frequency", main="")
