# Supplementary material for: Exploring Vietnamese co-authorship patterns in social sciences with basic network measures of 2008-2017 Scopus data
Source: F1000Res. 2017 Aug 24;6:1559. [Version 1] doi: 10.12688/f1000research.12404.1 (PMC5590089; doi:10.12688/f1000research.12404.1)
Supplement: Peer review history

# Peer review: Referee response for version 1

**DOI:** 10.5256/f1000research.13433.r25359

**Published:** 2017-09-29

**Authors:** Ly Thi Tran

**Affiliations:** 1 School of Education, Deakin University, Geelong, Vic, Australia

This interesting and important paper addresses the nature and structure of the network of Vietnamese social scientists who have published in Scopus-indexed journals in the period of 2008–2017. This is a critical issue to Vietnam given the government’s recent emphasis on enhancing research capacity and scientific outcomes. The topic of networking and co-authorship is very timely given Ministry of Education and Training’s new policies requiring doctoral candidates to publish in Scopus and Web of Science-indexed journals, as a compulsory component of their PhD.

 Overall, the paper is nicely written. The literature review provides an effective overview of the status of network statistics analysis and makes reference to the broader international context as well as the Asia region. The aims of the research are clearly articulated. There is a good balance between the presentation and discussion of the data. Even though I do not have statistical expertise and am unable to comment on the detailed analysis, generally I found the key findings of the research logically presented and easy to follow.

 A key strength of the paper lies in the way the authors use social network analysis to interpret and explain Vietnamese social scientists’ research performance. Based on this analysis framework, interesting insights into the structure of the network, the level of connection as well as the level of clustering in the network have been discussed.

 I have five comments below for the authors to consider in enhancing this research project or expanding this research area:

1. More discussion of **the specific implications of this research for improving research capacity and doctoral education** would be very useful in the current context of Vietnam. This information would be welcome by policy makers, leaders of universities and research institutes as well as research team, individual researchers and doctoral candidates. Given the scope of this paper, I hope a follow-up article may focus more on this aspect.
2. A critical issue that should be further explored is what facilitates or inhibits **productive** and **sustainable** networks among the researchers rather than just an one-off or brief collaboration or co-authorship?
3. The authors mainly focus on using social network analysis to explain research performance but less on predicting research performance and impact. Perhaps a follow-up study can address this issue and may include interviews/survey with the scientists to provide more nuanced understandings about the topic. An issue of great concern is what characteristics and structure of networks are likely to lead to **not only research productivity but also research impact**.
4. A further issue is whether **the network facilitated at the institutional/organisational level or at the individual researcher level is more sustaining and productive**? In this regard, what might be the incentives or support mechanism from the institution and government (for example, targeted funding for network/partnership development or grant/funding scheme in which capacity for research collaboration or network is one of the assessment criteria) needed?
5. One of the findings of this study is that “the network shows the potential of an intellectual elite composed of well-connected, productive, and socially significant individuals.” What is the implication of this finding for policy makers and education leaders to **facilitate an equitable and inclusive networking and collaboration structure **that supports rather than marginalises less established or ‘non-elite’ Vietnamese researchers including early career researchers, researchers from regional universities and researchers who were not exposed to overseas education.

In sum, I have enjoyed reading this paper and I congratulate the authors for this valuable work! Thank you for the opportunity to read and comment on this paper.

I have read this submission. I believe that I have an appropriate level of expertise to confirm that it is of an acceptable scientific standard.

# Peer review: Referee response for version 1

**DOI:** 10.5256/f1000research.13433.r25518

**Published:** 2017-09-07

**Authors:** Donaldine E . Samson

**Affiliations:** 1 Hawaii Pacific University, Honolulu, HI, USA

Given my knowledge about Vietnam's education system, publishing experiences, I have found the paper's results compelling and cogent. This represents one of the first attempts in Vietnam to study its social sciences research efforts from the community and collaboration perspectives.

 The authors intentionally employ network data analysis to explain what cross-section data could hardly tell. Scientific soundness of this research is based on earlier work on social networks and basic network metrics. The authors use a precise and unbiased definition of connectedness based on Newman’s study ^1^. The network visualization tools are not only original in this thread of research, they are especially useful in facilitating understanding of the statistical analysis. The paper is well-structured, striking a balance between data and discussions. The results are clear, easy to understand, and presented logically. Despite the focus on Vietnam social science researchers, the approach can also be productively applied to other researcher populations. 

 This study of co-authorship patterns can be used to help researchers and research directors understand the structure of research collaboration in social sciences in Vietnam and thus develop procedures, platforms and incentives to increase the robustness of networks and reduce the risk of networks disintegrating with the departure of a highly-connected individual researcher.

 The authors identify the potential emergence of an “intellectual elite.” More study on the effect of this group and ways to increase the benefits and reduce the risks would be interesting as it is not clear whether this intellectual elite is detrimental or beneficial to the maturation of a robust social science research community in Vietnam.

 I sincerely hope that the authors will continue this direction of research in social network analysis and look forward to their new results.

I have read this submission. I believe that I have an appropriate level of expertise to confirm that it is of an acceptable scientific standard.

## References

[1] NewmanME: Scientific collaboration networks. I. Network construction and fundamental results. *Phys Rev E Stat Nonlin Soft Matter Phys*.2001;64(1 Pt 2) : 10.1103/PhysRevE.64.016131 016131 10.1103/PhysRevE.64.016131 11461355

# Peer review: Referee response for version 1

**DOI:** 10.5256/f1000research.13433.r25520

**Published:** 2017-09-05

**Authors:** Tuyen Quang Tran

**Affiliations:** 1 Vietnam National University, University of Economics and Business, Hanoi, Vietnam

As a member of the evaluation council on economic studies for Vietnam’s National Foundation for Science and Technology Development (NAFOSTED), I am particularly interested in this research as this is one of the key issues we have been facing in recent years. Its merits can also be seen immediately. Some of my comments follow.

 The authors have shown their heroic act in collecting and preparing the unique data sets at individual levels, which has never been done before.

 The insights are clear and useful, with potentially practical implications for policy makers like us. I like the fact that they focus on basic measures where and when complexities do not necessarily bring more useful understanding.

 All data sets are presented, accompanied by R codes, which have made the replication and reproduction of the results both easy and transparent. Having gone through the paper several times, I am now pleased to approve this work.

 A further comment: for more valuable analyses in the future regarding this theme of research, I would suggest the expanding of the current data sets to include Scopus citation data. 

 I would like to suggest that some more limitation regarding the sources of data and methods. For example, ISI web of science covers less journals than Scopus, or panel data should be better for future research.

 I wish the authors every success in their future research attempt.

I have read this submission. I believe that I have an appropriate level of expertise to confirm that it is of an acceptable scientific standard.
